# Dinoflagellate Host Chloroplasts and Mitochondria Remain Functional During *Amoebophrya* Infection

**DOI:** 10.3389/fmicb.2020.600823

**Published:** 2020-12-18

**Authors:** Ehsan Kayal, Catharina Alves-de-Souza, Sarah Farhat, Lourdes Velo-Suarez, Joanne Monjol, Jeremy Szymczak, Estelle Bigeard, Dominique Marie, Benjamin Noel, Betina M. Porcel, Erwan Corre, Christophe Six, Laure Guillou

**Affiliations:** ^1^Fédération de Recherche 2424 Sorbonne Université & Centre National pour la Recherche Scientifique, Station Biologique de Roscoff, Roscoff, France; ^2^Algal Resources Collection, Center for Marine Sciences, University of North Carolina Wilmington, Wilmington, NC, United States; ^3^Génomique Métabolique, Génoscope, Institut François Jacob, CEA, CNRS, Université d’Evry, Université Paris-Saclay, Evry, France; ^4^UMR 1078, Genetics, Functional Genomics and Biotechnology, INSERM. UFR Médecine, Brest, France; ^5^UMR 7144 Sorbonne Université & Centre National pour la Recherche Scientifique, «Adaptation and Diversity in Marine Environment», Team «Ecology of Marine Plankton, ECOMAP», Station Biologique de Roscoff, Roscoff, France

**Keywords:** parasitism, dinoflagellate, organelles, *Amoebophrya*, marine plankton, chloroplast, kleptoplast

## Abstract

Dinoflagellates are major components of phytoplankton that play critical roles in many microbial food webs, many of them being hosts of countless intracellular parasites. The phototrophic dinoflagellate *Scrippsiella acuminata* (Dinophyceae) can be infected by the microeukaryotic parasitoids *Amoebophrya* spp. (Syndiniales), some of which primarily target and digest the host nucleus. Early digestion of the nucleus at the beginning of the infection is expected to greatly impact the host metabolism, inducing the knockout of the organellar machineries that highly depend upon nuclear gene expression, such as the mitochondrial OXPHOS pathway and the plastid photosynthetic carbon fixation. However, previous studies have reported that chloroplasts remain functional in swimming host cells infected by *Amoebophrya*. We report here a multi-approach monitoring study of *S. acuminata* organelles over a complete infection cycle by nucleus-targeting *Amoebophrya* sp. strain A120. Our results show sustained and efficient photosystem II activity as a hallmark of functional chloroplast throughout the infection period despite the complete digestion of the host nucleus. We also report the importance played by light on parasite production, i.e., the amount of host biomass converted to parasite infective propagules. Using a differential gene expression analysis, we observed an apparent increase of all 3 mitochondrial and 9 out of the 11 plastidial genes involved in the electron transport chains (ETC) of the respiration pathways during the first stages of the infection. The longer resilience of organellar genes compared to those encoded by the nucleus suggests that both mitochondria and chloroplasts remain functional throughout most of the infection. This extended organelle functionality, along with higher parasite production under light conditions, suggests that host bioenergetic organelles likely benefit the parasite *Amoebophrya* sp. A120 and improve its fitness during the intracellular infective stage.

## Introduction

Many parasites infecting unicellular eukaryotes are necrotrophs, i.e., they kill their host before digesting them. This strategy prevents the host from escaping by evolving resistance mechanisms during the infection process. A notable exception is found in the widespread marine biotrophic parasites of the order Syndiniales (Dinoflagellata), which appear to avoid killing their hosts during most of their intracellular developmental stages, meaning that the host is digested “alive,” i.e., while maintaining a certain degree of physiological functions. Among them, *Amoebophrya* spp. (Amoebophryidae or MALV-II clade) are obligate unicellular endoparasites infecting a wide range of marine planktonic protists, including other dinoflagellates as well as other Syndiniales (Park et al., 2004).

*Amoebophrya* spp. appear to be pervasive components of the marine planktonic ecosystems (Vargas et al., 2015) and of particular interest for their potential role to controlling populations of bloom-forming toxic dinoflagellates (Chambouvet et al., 2008). A typical infection cycle lasts 2–3 days with the entire consumption of the host cellular content by the parasite, followed by the release of parasitic infective propagules called dinospores (Cachon, 1964; Coats and Park, 2002). Interestingly, dinoflagellate cells infected by *Amoebophrya* spp. maintain a degenerating but steady swimming behavior until the release of dinospores. While such swimming behavior is well-known to those who work with *Amoebophrya* cultures and has also been observed in the wild (personal observations), it has only been reported once in the literature (Park et al., 2002a). The decline in the swimming speed of the infected dinoflagellate host has been linked to lower energy availability, likely resulting from the disruption of cellular functions (Park et al., 2002b). However, it is not clear whether this lower energy availability is the result of the degradation of the cell-machinery (following the consumption of the host cell) and/or the concomitant energy uptake by the developing parasite.

In most eukaryotes, energy is primarily produced by one or two organelles, namely the chloroplast and the mitochondrion. These organelles maintain highly reduced genomes, mainly encoding key energy-related genes and some means for their expression (Allen, 2015). Dinoflagellate plastidial and mitochondrial genomes give an eloquent example of extreme gene content reduction, as they only encode a tiny fraction of the genes required for their activity: the mitochondrion only encodes 3 proteins and 2 partial ribosomal genes, and 12 proteins and 2 ribosomal genes are present in the chloroplast (Smith and Keeling, 2015; Barbrook et al., 2019). Thus, these bioenergetics organelles are highly dependent upon the expression of nucleus-encoded genes and any disruption of this activity is expected to greatly affect their functioning. Interestingly, while some *Amoebophrya* strains start their development in the host cytoplasm (hereafter referred as cytoplasmic strains), others primarily target and digest the host nucleus (intranuclear strains) (Coats and Park, 2002), likely resulting in the knockout of the host nuclear machinery at the beginning of the infection. By comparing the influence of cytoplasmic and intranuclear infections on photosynthesis in two strains of dinoflagellates, Park et al. (2002b) showed that nucleus digestion by the intranuclear parasite strain resulted in far greater disruption of the chloroplast photosynthetic activity in the host compared to the cytoplasmic strain. However, the mechanisms involved in the disruption of the host bioenergetics processes are not yet understood. For instance, it is unclear whether the host organelles remain functional through the infective cycle associated with intranuclear parasitic development and, if that is the case, whether the parasite actually benefits from the products of the host organelles.

Here, we report the effects of infections by the intranuclear *Amoebophrya* sp. strain A120 (belonging to the MALVII clade, cluster 2, subcluster 4; Cai et al., 2020) on key organellar functions of the dinoflagellate *Scrippsiella acuminata*. Our results showcase functional host chloroplasts up to the final stages of the infection along with higher dinospore production, i.e., the amount of host biomass converted to parasite infective propagules, when exposed to constant light compared to dark conditions. Moreover, by analyzing transcriptomic data throughout a full infection cycle, we show differential expression patterns of genes involved in the host organellar energy-related metabolic pathways compared to nuclear counterparts.

## Materials and Methods

### Origin of Strains and Culture Conditions

Both host and parasitic strains were isolated from the Penzé Estuary (North-West of France, English Channel; 48°37′N, 3°56′W). The culture of *Amoebophrya* sp. strain A120 was established by manually isolating a swimming infected dinoflagellate host (recognized thanks to the natural bright-green auto-fluorescence of the parasite) collected in the field using an epifluorescence microscope Olympus BX51 equipped with the U-MWB2 filter (excitation 450–480 nm, emission 500 nm; Coats and Bockstahler, 1994). The infected host was then transferred to and maintained in exponentially growing cultures of the non-toxic dinoflagellate *Scrippsiella acuminata* (previously known as *S. trochoidea*, Kretschmann et al., 2015) that was established from the germination of a single cyst collected from sediments in 2005 (strain ST147). Both strains are accessible at the Roscoff Culture Collection website^1^ with the following accession numbers: ST147 = RCC1627 for *S. acuminata* and A120 = RCC4398 for the *Amoebophrya*-like parasite.

We used continuous light in order to minimize circadian variations of the host metabolism, such as pigment cell content and composition. We have found that continuous light does not seem to stress phytoplanktonic organisms, as illustrated by significant increases in growth rates under this condition (data not shown). Consequently, *S. acuminata* cultures were acclimated to continuous white light in exponential growth phase under 85 μmol photons m^–2^ s^–1^ for over 3 weeks prior to the experiments. We used fluorescent tubes (Sylvania Luxline plus) and adjusted the irradiance using a quantamer (Biospherical Instrument Inc.) and neutral density filters (LEE filters). The cultures were grown at 22°C in vented flasks (Culture One) filled with natural seawater from the Penzé Estuary complemented with F/2 medium (Sigma Aldrich) and 5% soil extract (Chambouvet et al., 2008).

### Experimental Design

We performed two independent experiments of infection of *S. acuminata* ST147 by *Amoebophrya* sp. A120, mainly differing by the initial parasite:host ratio and sampling times. Despites the differences in ratios, we observed massive infections for both experiments corresponding to 100% prevalence (see below). In both experiments, the free-living infective stage of the parasite (the dinospores) was freshly prepared from several infected *S. acuminata* cultures and harvested by gentle separation from the remaining host cells using gravity filtration through 5 μm polycarbonate filters (Whatman).

The first experiment (hereafter called “experiment 1”) was used to investigate the influence of light on the development of *Amoebophrya* sp. A120, as well as the photosynthetic activity and the pigment composition of the infected *S. acuminata* ST147 host. We also estimated the effect of light on parasite production, which was assessed based on the conversion of host to parasite biomass (i.e., carbon content). For that, hosts and parasites were mixed in 50 mL culture flasks with an initial dinospore:host ratio of 50:1 and placed either under light or dark conditions, each in four replicates (host and dinospore countings are detailed below). Flasks containing uninfected hosts from the initial cultures were similarly sampled during the experiment and used as control. In order to estimate host and dinospore cell density, we performed 11 samplings of both infected and control cultures over a 94 h period, and for 6 of those we estimated the pigment composition. In addition, parasite prevalences were estimated after 40 h of incubation to check for the effect of light conditions on parasite infectivity.

The second experiment (hereafter “experiment 2”) was used for transcriptomics analyses and has been previously described by Farhat et al. (2018). Briefly, 21 culture flasks (600 mL) were inoculated by mixing 150 mL of dinospores of *Amoebophrya* sp. A120 and 300 mL of exponentially growing *S. acuminata*. ST147 (dinospore:host ratio of 30:1) cultures. Every 6 h, three flasks were completely harvested (7 times from 0 to 36 h) for mRNA extraction and sequencing, as detailed in Farhat et al. (2018). Three flasks containing 450 mL of uninfected hosts were additionally prepared to be used as control and completely harvested at 0 h (T0). Before each harvest, infected cultures were sampled in order to estimate the prevalence of the parasite.

### Infection Dynamics

#### Host and Dinospore Counts

In experiment 1, samples (1.5 mL) were fixed with 0.25% glutaraldehyde (Grade II, Sigma Aldrisch) and stored at −20°C before analysis. Dinospores have been stained with SYBR Green-I (1/5,000th final concentration from commercial stock) during 30 min before flow cytometry analysis. In both cases, cell counting was performed using a FACS Canto flow cytometer (Becton Dickinson) equipped with a 488 nm laser and standard filter setup (Marie et al., 2000). We detected host cells by their natural chl *a* red fluorescence, while parasite dinospores were identified based on the fluorescence originating from their SYBR-Green stained DNA and the side-scatter parameter (SSC).

#### Parasite Prevalence

We estimated parasite prevalence by Fluorescence *in situ* Hybridization with Tyramide Signal Amplification (FISH-TSA). To do so, a 5 mL culture volume was sampled, then fixed in 1% paraformaldehyde (Sigma) and incubated for 1 h at 4°C in the dark, then filtered on polycarbonate filters (3 μm; Ø 25 mm) using a vacuum pump (<200 mm Hg). Following ethanol dilution series (50, 80, and 100%; 3 min each), dehydrated filters were briefly dried at room temperature before storage at −20°C until analyses. We used the oligonucleotide probe ALV01 (Syndiniales, MALV Group II) with a 5′ end aminolink (C6; MWGBiotech AG) labeled with horseradish peroxidase (HRP) to target the *Amoebophrya* infective stages inside the host (as described in Siano et al., 2011). Filters were manually counted under an Olympus BX-51 epifluorescence microscope (Olympus Optical) equipped with a mercury light source, a 11012v2-Wide Blue filters set (Chroma Technology, VT, United States), and a CCD camera (Spot-RT, Diagnostic Instrument, Sterling Heights, MI, United States), with fluorescence filter sets for propidium iodide labeling the nucleus (excitation: 536 nm; emission: 617 nm) and green autofluorescence generated by the probe (excitation: 495 nm; emission: 520 nm). Cells were counterstained with calcofluor (100 ng ml^–1^) for visualization of dinoflagellate theca. We estimated the parasite prevalence by averaging infection counts on a minimum of 50 host cells.

#### Carbon Content Estimation

We used the cell biovolume (V) as a proxy for the carbon content of both host and parasite based on the dimensions and geometric shape of their cells (i.e., prolate spheroid; Hillebrand et al., 1999) followed by carbon estimation using the equation proposed by Menden-Deuer and Lessard (2000) for *S. acuminata* [e.g., pgC cell^–1^ =−0.119 + 0.819 × log V (μm^3^)] and the conversion factor of 100 fg C μm^–3^ proposed by Børsheim and Bratbak (1987). Parasite production (i.e., percentage of host carbon biomass converted to dinospores) was estimated by comparing dinospore and host carbon contents at 88 h and 24 h, respectively.

### Host Physiology

#### Photosynthetic Activity

The quantum yield of photosystem II (PSII; F_V_/F_M_) was measured with a Pulse Amplitude Modulation (PAM) fluorimeter (PHYTO-PAM I, Walz, Germany). The basal fluorescence level (F_0_) was measured upon excitation by 450 nm modulated inactinic light after 10 min relaxation in darkness, while the maximum fluorescence level in the dark acclimated samples (F_M_) was determined after triggering a saturating light pulse (655 nm, 4,000 μmol photons m^–2^ s^–1^, 400 ms). We calculated the maximal PSII fluorescence quantum yield using the formula F_V_/F_M_ = (F_M_ – F_0_)/F_M_.

#### Pigment Composition

The natural chl *a* fluorescence of *Scrippsiella* cells was measured by flow cytometry (simultaneously to cell counts explained above) and estimates were normalized to the fluorescence of 3 μm standard fluorescent YG beads (Polysciences Warring- ton, PA, United States). The composition in other pigments was determined by High-Pressure Liquid Chromatography (HPLC) using a Helwett Packard 1100 system following Nézan et al. (2014). We detected chlorophyll *c* and carotenoid pigments by their absorbance at 440 nm using diode-array spectroscopy and identified based on their absorption spectra and retention time (Roy et al., 2011).

### Transcriptomics

#### Host Transcriptome and Proteome

We downloaded and reanalyzed a recently published transcriptomics dataset from the internal development of two *Amoebophrya* strains (ENA database project ID PRJEB26803) following a full infection cycle (Farhat et al., 2018). We reconstructed the host organellar energy-related metabolic pathways for which part of the genes are encoded by either the mitochondrial (mt) or the chloroplast (cp) DNA, and monitored their expression. To do so, a reference transcriptome (Step 1 in the bioinformatics workflow; Supplementary Figure S1) was created for the host *S. acuminata* (host-T0) from reads of the uninfected host using the Trinity assembler v.2 (Grabherr et al., 2011). We created a reduced version of this reference transcriptome using companion scripts from the Trinity package (Haas et al., 2013) by filtering out poorly covered transcripts (FPKM < 1) and rare isoforms (<1%), then predicted a proteome (host-T0p) with TransDecoder v.3.0.1^2^. Both the reference transcriptome host-T0 and the predicted proteome host-T0p were automatically annotated using a combination of similarity searches with Diamond v.0.8.34 (Buchfink et al., 2015) against the UniProtKB/Swiss-Prot (version October 2019) and the GenBank nr (version October 2019) databases, hmmscan from HMMER v.3.1b2^3^, SignalP v4.1 (Petersen et al., 2011), and TMHMM v2.0^4^. Finally, GO terms annotation and KEGG mappings were performed and all the annotations were integrated with the Trinotate pipeline^5^.

#### Manual Curation of Energy-Related Genes

We used the KEGG Pathway reference database^6^ to identify all recorded genes whose products are involved in the electron transport chains (ETC) of the “Oxidative phosphorylation” or OXPHOS (ko00190) and the “Photosynthesis” (ko00195 and ko00196) pathways, and searched for them in our transcriptome and proteome annotations (Step 2 in the bioinformatics workflow; Supplementary Figure S1). For each gene, a set of reference protein query sequences was downloaded from the UniProtKB/Swiss-Prot database (UniProt Consortium, 2019) and used as queries in similarity searches with the blastp and tblastn scripts (*e*-value = 1e^–10^) from the BLAST package v2.6 (Altschul et al., 1990, 1997) to capture homologs from the predicted proteome (host-T0p) and reference transcriptome (host-T0) of *S. acuminata*. We checked positive hits by reverse BLAST against the UniProtKB/Swiss-Prot database. A combination of gap4 v.4.11 (Staden et al., 2000) and gap5 (Bonfield and Whitwham, 2010) from the STADEN package^7^, MITObim v.1.8 (Hahn et al., 2013), Bowtie2 v2.2.9+ (Ben and Salzberg, 2012), and scripts from the Samtools v.1.3.1 package (Li et al., 2009) were used to manually curate and extend positive matches. For each gene, we used the ExPASy Translate tool from SIB ExPASy Bioinformatics Resources Portal (Artimo et al., 2012)^8^ with the Standard Genetic Code to translate the selected transcripts into predicted peptides. Gene transcripts were retained when predicted peptide matched homologs on public databases (blastx using the Standard Genetic Code against nr and the UniProtKB/Swiss-Prot databases), and preferably included both a start and a stop codon with a portion of the untranslated regions (UTR) at the 5′ and 3′ ends of the CDS region (excluding those genes encoded by organelle genomes). Finally, we used a phylogenetic approach to confirm the identity of the selected genes as follows. Translated peptides were aligned to homologs from the reference sets using the MAFFT v7 (Katoh et al., 2019) aligner with default parameters and the most variable sites were removed using Gblocks v0.91b (Castresana, 2000) except for highly divergent sequences where unfiltered alignments were retained. We reconstructed single gene phylogenetic trees for each alignment using RAxML v8 (Stamatakis, 2014) with the “auto” switch and individually visualized each tree using FigTree v1.4.3^9^. The presence of transit and signal peptides were predicted with the TargetP v.2.0 (organism groups: Plants), and SignalP v.5.0 (Armenteros et al., 2019) from the DTU Heakth Tech online server^10^. The reconstructed ETCs of OXPHOS and the light phase of photosynthesis in *S. acuminata* are shown in Supplementary Figures S3, S4, respectively.

#### Transcriptomics of Infection

We created a modified transcriptome for the host (host-T0-new) by identifying and replacing contigs from the host-T0 transcriptome that matched the curated energy-related gene sets identified above (Step 3 in the bioinformatics workflow; Supplementary Figure S1) using tblastn (*e*-value = 1e^–5^). An artificial “hybrid” reference transcriptome (hybtrans) was created by combining the modified host transcriptome host-T0-new and the parasite predicted mRNAs (that include both the 5′ and 3′ UTR regions) for the *Amoebophrya* A120 (Farhat et al., 2018). We used the DESeq2 differential expression analysis tool from the Trinity v2 package (Haas et al., 2013) and custom scripts to monitor the abundance of host and parasite genes in hybtrans throughout the infection. In short, filtered RNA-seq reads for each replicate (triplicates sampled every 6 h of a 36 h infection cycle in experiment 2) were separately mapped with the Bowtie2 aligner, and gene expression matrices were computed using the RSEM method. Not cross-sample normalized transcript per million (TPM) values were calculated for each species, and each time step separately. All scripts and gene sequences used in this study are available on the *Amoebophrya* genome website^11^.

### Statistical Analysis

All data were transformed [log (*x* + 1)] before analysis using the basic package “stats” in the R software (R Core Team)^12^. For experiment 1, we used Mann-Whitney tests to compare dinospore production and host photosynthetic activities at each sampling time (*N* = 4) between light and dark conditions in both infected and uninfected treatments. The same test was used to compare the conversion of host to dinospore biomass under light and dark conditions (*N* = 4). To compare temporal patterns of gene expression in experiment 2, we used polynomial regressions to fit no-linear relationships between time (hours) and TPM (*N* = 21). In all polynomial regression analyses, we used 3-degrees polynomial features given they resulted in the best significant fit.

## Results

### Effect of Light on Host Growth and Parasite Production

**FIGURE 1:**
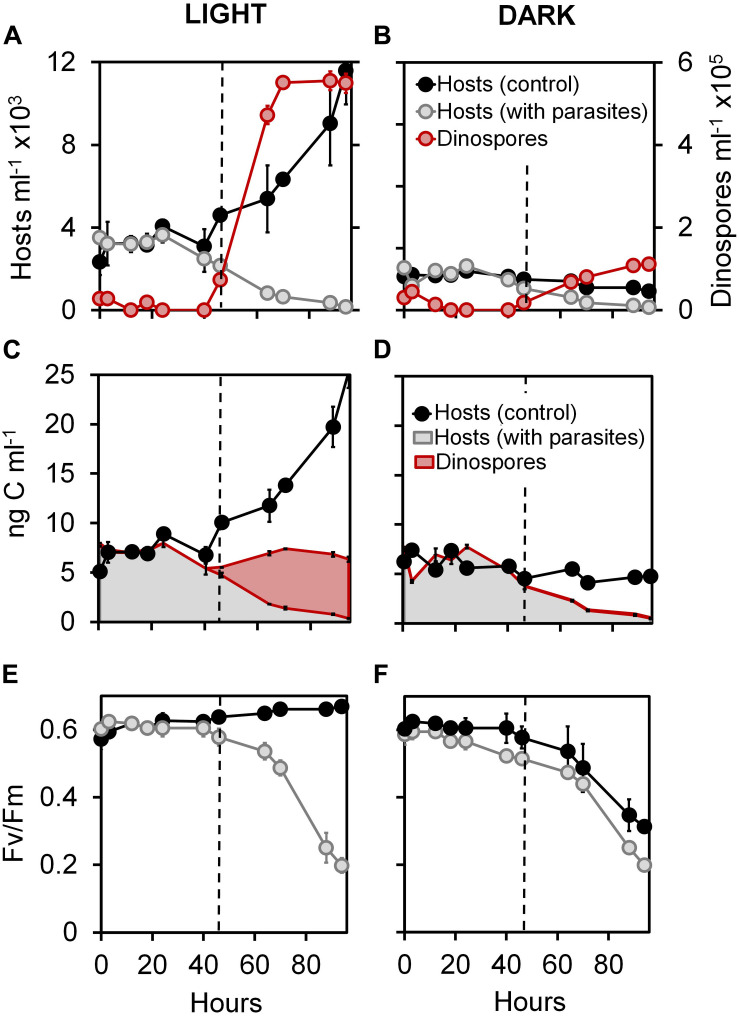
Effect of light on infection dynamics of the phototrophic dinoflagellate *Scrippsiella acuminata* and its photosynthetic activity by the Syndiniales *Amoebophrya* sp. A120. Variations in the cell densities of **(A,B)** and biovolume-inferred biomass associated to **(C,D)** host cell and parasite dinospores in cultures under continuous light **(A,C)** or dark **(B,D)** conditions. Evolution of the quantum yield of photosystem II (F_V_/F_M_) measured during infection of ST147 by A120 under continuous light **(E)** or dark **(F)** conditions.

Host cell densities were nearly identical between healthy (control) and infected cultures under both light and dark conditions (Mann-Whitney test, *p* > 0.01) for the first 40 h of experiment 1, followed by a growth spurt in the uninfected treatments at 74 h under light conditions (net growth rate of 0.3 d^–1^) and decrease in infected treatments (Figures 1A,B). Infection prevalence (79 and 75% after 40 h under light and dark conditions, respectively) and duration of the parasite internal development (46 h followed by bursts of dinospore) were similar in infected cells independent of the light condition (Mann-Whitney test, *p* = 0.23). Interestingly, we observed that the dinospore density was 5-fold lower at the end of the experiment in dark compared to light conditions (Mann-Whitney test, *p* = 0.03). Moreover, net conversions of the available host biomass (maximum of density estimated at 24 h) into dinospore biomass (maximum of density estimated at 88 h) were 75.62 and 14.77% (Figures 1C,D) in light and dark conditions, respectively (Mann-Whitney test, *p* = 0.002).

### Host Photosynthetic Activity During Infection

At the beginning of experiment 1, the photosystem II of *S. acuminata* chloroplasts displayed near-optimal quantum yield values (F_V_/F_M_ = 0.6_;_ Mann-Whitney test, *p* < 0.01) in all cultures (Figures 1E,F). In light conditions, the F_V_/F_M_ parameter of the infected culture were similar (Mann-Whitney test, *p* < 0.01) to those of the uninfected control during the first 46 h (Figure 1E), corresponding to the intracellular stages of the parasite life cycle. The photosystem II quantum yield remained constant in the controls and dropped in the infected cultures only after 46 h. By comparison, this parameter remained high until 24 h and then progressively decreased in both control and infected cultures under constant darkness (Figure 1F).

We measured the natural chl *a* cell fluorescence of *S. acuminata* cells by flow cytometry in the light condition experiments (Supplementary Figure S2A). The infected cells show similar values of chl *a* fluorescence when compared to the control during the first 46 h of infection (i.e., until the first release of dinospores in the medium) followed by a sharp drop in chl *a* fluorescence consecutive to the collapsing of the host cell density.

In order to identify possible modifications of light utilization capacities in the host chloroplasts during the infection, we monitored the variations of the thylakoidal accessory pigments relative to chl *a* under constant light (Supplementary Figures S2B–F). *S. acuminata* ST147 displayed a pigmentation profile typical of peridinin containing dinoflagellates (Roy et al., 2011). The relative content in chl *c*_2_ (Supplementary Figure S2B) and the light-harvesting xanthophyll peridinin (Supplementary Figure S2C) remained stable throughout the infection cycle. We recorded similar relative contents of the photoprotective pigments β-carotene, diadinoxantin, and diatoxanthin between the infected and the healthy cultures throughout the infection (Mann-Whitney test, *p* < 0.01; Supplementary Figures S2D–F).

### Host Gene Expression During the Infection Cycle

#### General Patterns of Infection

**FIGURE 2:**
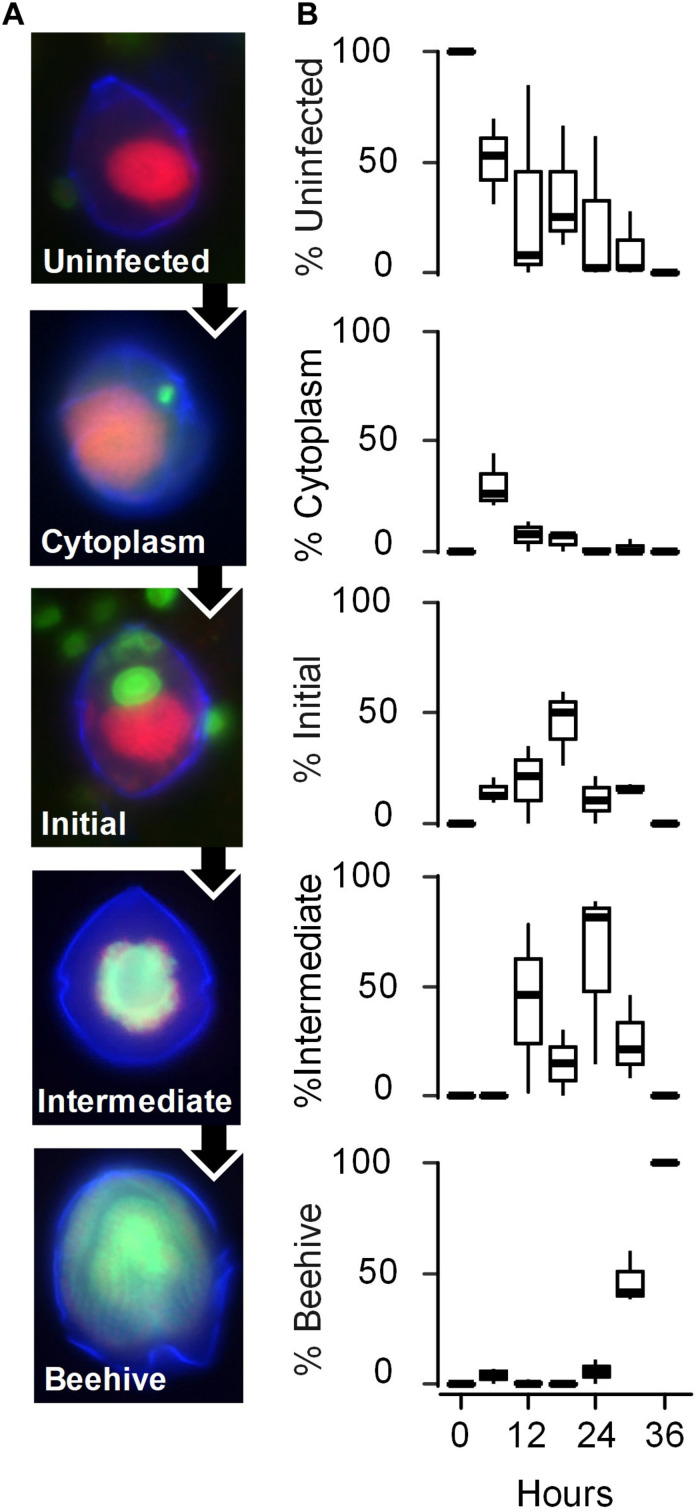
Evolution of the infection of the phototrophic dinoflagellate *Scrippsiella acuminata* ST147 by the Syndiniales *Amoebophrya* sp. A120. **(A)** The stages of the parasite infective cycle detected by fluorescence *in situ* Hybridization (FISH) was divided into four intracellular stages: stage 1 (Uninfected); stage 2 (Cytoplasm) where the parasite has penetrated the host and is located in the cytoplasm; stage 3 (Initial) where at least one parasite is located in the still detectable host nucleus; stage 4 (Intermediate) where the host has lost its nucleus but part of the cytoplasm remains; stage 5 (late infection or “Beehive”) where the completely digested host is replaced by a multinucleated sporont within the intact theca. Cell nucleus (red), dinoflagellate theca (blue), and fluorescence of probe ALV01 targeting *Amoebophrya* SSU ribosomal RNA (green) are shown. **(B)** Prevalence of the infection (in%) on the different stages of infection monitored every 6 h by FISH in biological triplicates (horizontal lines represent averages).

*S. acuminata* ST147 cells infected by *Amoebophrya* sp. A120 consistently showed active swimming behavior until the final stage of infection (Supplementary Video S1). We used FISH-TSA to investigate the progression of infection every 6 h during an incubation period of 36 h (experiment 2). The progression of the infection was characterized by the physical location and stage of maturation of the parasite in the host cell as follows (Figure 2A): uninfected (corresponding to T0); cytoplasmic (the parasite has penetrated the host cytoplasm and is migrating to the nucleus); initial (the parasite starts its growth inside the host nucleus); intermediate (the host nucleus is entirely consumed); beehive or last stage of infection (a multinucleated sporont within the intact theca the host shell). We found that *Amoebophrya* sp. A120 starts its internal development in the nucleus of *Scrippsiella acuminata* ST147, which is totally consumed within 24 h (“intermediate” stage). While replicates displayed a certain level of variability in the progression of the infection, all host cells were infected (100% of prevalence) and the parasites were all at the late stage of infection after 36 h (Figure 2B).

#### Reconstruction of the Host Organellar ETCs

We assembled a reference transcriptome for the host that contained 322,673 transcripts and 400,339 Transdecoder-predicted peptides, with 105,075 of the latter having GO-term annotations (data not shown). By screening both the host T0 transcriptome and predicted proteome, we identified key genes involved in the electron transfer chains (ETC) of the mitochondrial oxidative phosphorylation (OXPHOS) pathway (Supplementary Figure S3 and Supplementary Table S1) and the chloroplastidial thylakoidal light phase of photosynthesis (Supplementary Figure S4 and Supplementary Table S2). The mitochondrial ETC in *S. acuminata* is composed of four major and several smaller membrane-bound complexes, with the notable absence of the canonical NADH dehydrogenase replaced by an alternative NAD(P)H (NDA) dehydrogenases and the presence of an alternative oxidase (AOX) that could act as final electron acceptor (Supplementary Figure S3). The succinate dehydrogenase (SDH) complex appears to contain only two (the iron and flavoprotein) out of the four canonical subunits. We also identified unusual ATP synthase subunits previously described in the apicomplex *Toxoplasma gondii*. Most of the protein genes (49) are encoded by the nucleus (nDNA), 24 of them harboring mitochondrial transit peptides at their N-terminus (Supplementary Table S1), excluding three conserved genes (*cob*, *cox1*, and *cox3*) encoded by the mitochondrial DNA (mtDNA).

The chloroplastidial ETC of *S. acuminata* ST147 is typical of peridinin-containing chloroplasts: several proteins involved in light-harvesting antennas and their protection, the two photosystems (PSI and II), a cytochrome b6/f (CytB_6_f), and an ATP synthesis complexes (Supplementary Figure S4). We identified 11 predicted plastidial (cpDNA) and 72 nuclear-encoded protein genes (including 34 fucoxanthin-chlorophyll *a*/*c* binding protein-like isoforms), 28 of which carried signal peptides and/or transit peptides at their N-terminus (Supplementary Table S2). We failed to identify a homolog for the very short (∼114 nt corresponding to 38 amino acids) photosystem II *psbI* gene.

#### Gene Expression of the Host

**FIGURE 3:**
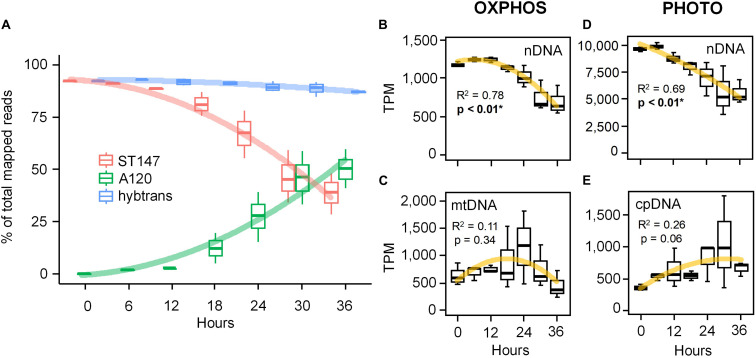
Gene expression patterns of the phototrophic dinoflagellate *Scrippsiella acuminata* ST147 infected by the Syndiniales *Amoebophrya* sp. A120 over a full 36 h infection cycle. **(A)** Evolution of the gene expression calculated as transcripts per million transcripts (TPM) not normalized across samples for both the host (ST147), the parasite (A120) and a concatenated “hybrid” reference transcriptome (hybtrans). Expression profiles of energy-related genes involved in the electron transfer chain (ETC) of both the mitochondrial OXPHOS **(B,C)** and the chloroplast photosynthesis **(D,E)** pathways in infected *S. acuminata* ST147 (see Supplementary Figures S3, S4 and Supplementary Tables S1, S2 for more details). Genes have been segregated based on their location: mtDNA, mitochondrion; cpDNA, chloroplast; nDNA, nucleus. Horizontal lines and error bars represent average and standard deviation, respectively.

After manual curation of energy-related genes, we built a modified transcriptome for the host (host-T0-new) consisting of 321,190 transcripts which, combined with the parasite transcriptomes, provided a meta-transcriptome (meta-T0) of 347,621 sequences. The average rate of mapped RNA-seq reads was similar for both the original (host-T0) and the modified (host-T0-new) transcriptomes (79–85% and 78–88%, respectively). Interestingly, despite prevalence of 100% (Figure 2B), 44% (*SD* = 10) of the total reads still mapped to the host transcriptome after 36 h of infection (Figure 3A). The differential expression (DE) analysis revealed 1,927 predicted genes to be overexpressed (DESeq2 method with 0.1 dispersion; *p*-value cutoff = 0.01 for FDR with a minimum of 2 × fold change). 437 of these genes had GO annotations mostly linked to metabolic (75%), biosynthetic (32%), and nucleobase-containing compound metabolic (27%) processes. However, the same lack of intra-replicate synchronization was evidenced in the DE analyses as observed in the prevalence (data not shown). We therefore focused our investigations on comparing the expression levels of genes involved in energy-related pathways (ETCs of OXPHOS and light phase of photosynthesis) in the infected host for which genes are located both in the nucleus (nDNA) and in organelles (cpDNA and mtDNA). Polynomial regression of TPM values against time indicated that nDNA genes for both OXPHOS (Figure 3B and Supplementary Figures S5A–D) and the light phase of photosynthesis pathways (Figure 3C and Supplementary Figures S6A–G) depicted a similar pattern of expression characterized by a decreasing sigmoid curve after 12 h of infection (*R*^2^ = 0.78 and 0.69, respectively, *p* < 0.01). By comparison, we observed an apparent increase in the expression of mtDNA (up to 24 h) and cpDNA (up to 36 h) genes, although not statistically significant (*R*^2^ = 0.11 and 0.26, respectively; *p* > 0.05, Figures 3D,E). All mtDNA (*cob*, *cox1*, and *cox3*; Supplementary Figures S5E–I) and 9 out of the 11 cpDNA (from complexes PSI, PSII, and Cytb_6_f; Supplementary Figures S6H–J) protein genes exhibited a unimodal pattern of gene expression. The expression of the three mtDNA genes peaked at 18–24 h, while those of the nine cpDNA genes were maximum at 24–30 h of infection (Figure 3). Surprisingly, two subunits of the plastidial ATP synthase (*atpA* and *atpB*) genes encoded by the cpDNA displayed the same pattern of expression as their nuclear counterparts (Supplementary Figure S6K).

## Discussion

### Host Plastidial Activity and Light Utilization by the Parasite

In this study, we monitored the physiological and genetic responses of the dinoflagellate *Scrippsiella acuminata* ST147 to infection by the Syndiniales *Amoebophrya* sp. A120, which primarily targets the host nucleus after entry into the cell. Our result show that the absence of light during the infection had no effect on the duration of the internal development nor the prevalence of the parasite. However, the lack of light negatively impacted the number of dinospores produced per infected host cell and the estimated biomass transfer yield from the host to the parasite (Figures 1A–D; Mann-Whitney test, *p* = 0.002).

The photosystem II (PSII) complex carries out the initial charge separations of the light phase of photosynthesis and is very sensitive to perturbation or stress, which trigger the photoinactivation process (see e.g., Takahashi and Murata, 2008). The quantum yield of PSII thus constitutes a useful parameter for monitoring the integrity of the chloroplastidial ETC under stress. Our results show that PSII complexes remain efficient in *S. acuminata* ST147 infected cells (Figure 1E), suggesting that the thylakoidal light phase of photosynthesis is quite functional during the parasite intracellular stages. These findings are corroborated by the resilience of cpDNA-encoded mRNAs when compared to their nuclear counterparts (Figures 3D,E). In addition, it is well-known that a number of β-carotene molecules are bound to the reaction center of PSII and act as sink for singlet oxygen (Telfer, 2005; Ishikita et al., 2007). The absence of significant β-carotene degradation and phaeopigment induction as a result of oxidative stress, along with the stability of both light-harvesting and light-dissipating pigments of the photosynthetic antennae together reflect consistent light utilization by the chloroplast during the infection. These observations strongly suggest that infection of *S. acuminata* ST147 by *Amoebophrya* sp. A120 does not induce any significant photophysiological stress in the host chloroplast. Instead, the chloroplast of infected *S. acuminata* ST147 likely maintains steady ATP and NADPH production in the plastidial stroma during the whole duration of the internal development of the parasite.

While the dinoflagellate host appears to be “alive,” i.e., continued swimming, intracellular infection will rapidly alter most of its physiological functions, following a modality that seems to differ depending whether the parasite first targets the host cytoplasmic or nucleus content. Previous studies reported that the dinoflagellate *Levanderina fissa* (formerly *Gyrodinium instriatum*) infected by a cytoplasmic strain of *Amoebophrya* sp. maintains plastidial carbon fixing activity throughout infection, suggesting that the Calvin-Benson cycle remained functional (Park et al., 2002b). By contrast, in the dinoflagellate *Akashiwo sanguinea* infected by an intranuclear strain, carbon fixation was impaired from the early stages of the infection, with pronounced degradation of chl *a*. The authors linked the chloroplast shutdown in *A. sanguinea* to the digestion of the nucleus by the parasite, whereas the intact nucleus of *L. fissa* would sustain chloroplast functions through the uninterrupted synthesis of nuclear-encoded photosynthetic proteins (Park et al., 2002b). In our study, infection by the intranuclear *Amoebophrya* sp. A120 did hinder photosynthetic functions in *S. acuminata* ST147 (Figure 1E). Considering the short timespan of the parasite intracellular development period (36 h under the experimental conditions of this study), it is likely that the chloroplast of *S. acuminata* was quite capable of relying upon the existing pool of nuclear-encoded photosynthetic proteins in order to maintain plastidial activity despite the early digestion of its nucleus. This suggests the existence of a variety of physiological infective strategies among *Amoebophrya* species.

Considering that during the first 46 h of the first experiment (1) the host density remained constant under dark condition; (2) no drastic difference in host density was observed between the light and the dark conditions; (3) the host chloroplasts remained functional under dark condition, we therefore conclude that non-infected hosts survived the first 46 h of darkness, likely in lower physiological conditions compared to the cells under light. While we cannot clearly disentangle the various effects of the dark conditions on the host physiology, our results strongly suggest that light, while not essential, is an important factor for the fitness of *Amoebophrya* sp. A120. This is in line with previous observations suggesting that environmental factors such as water quality and nutrient levels impact spore productivity and infectivity (Yih and Coats, 2000), whereas the duration of the internal development of the parasite is dependent upon the host-parasite system (Park et al., 2013). A major limitation of our study concerns its reliance on indirect evidence of kleptoplastidy by the parasite. Such shortcoming can be addressed by the use of stable isotopes of inorganic carbon (i.e., HCO${}_{3}^{-}$), only accessible to the photosynthetic host but not to a heterotrophic parasite, on infected cultures and monitor whether ^13^C has been incorporated by the dinospores.

### mt-mRNAs as a Proxy for Functional Organelles During Infection

Morey and Van Dolah (2013) reported that mRNA half-lives were substantially longer for genes involved in housekeeping processes, including energy metabolism and transport, whereas genes involved in organellar post-transcriptional regulation belonged to the pool with the shortest half-life. *Amoebophrya* sp. A120 primarily digests the host nucleus, thereby knocking out the main gene expression machinery of the host. Consequently, the apparent upregulation of genes related to information processing and cell cycle processes (Supplementary Figure S7) in our DE analyses could be explained by the longer half-lives rather than by the *de novo* synthesis of these mRNAs. However, our results feature stark differences in the rarefication rate of mRNAs for the same pathways (mitochondrial and plastidial ETCs) based on the location of those genes. For instance, while the expression of nuclear-encoded ETC genes steadily decreased throughout the infection, all mitochondrial (mtDNA) and most (9 out of 11) chloroplastidial (cpDNA) encoded protein genes displayed an apparent peak of expression at 18–24 h and 24–30 h, respectively (Figures 3D,E; Supplementary Figures S5E–I and Supplementary Figures S6H–J). One explanation for the apparent increase in mt and cp-mRNAs can involve the presence of a residual pool of RNA polymerases. Moreover, the degradation of organellar mRNAs is delayed (longer effective half-lives) compared to those from the nucleus as it relies solely on ribonucleases (RNase) present in the organelles while being protected from RNases from the parasite by intact mitochondrion and chloroplast membranes. Altogether, our results strongly suggest that the chloroplast and the mitochondrion remain active and might fuel the active swimming of the denucleated host during the internal stages of the infection (Supplementary Video S1), a behavior reminiscent of an undead “zombie” dinoflagellate. Active swimming has been previously observed in other dinoflagellates infected by *Amoebophrya* spp. (Park et al., 2002a) and might eventually increase the dispersion capacity of the dinospores and their probability to reach new hosts.

### A Novel Kind of Organellar Retention?

*Amoebophyra* sp. A120 is an aerobic phagotrophic endoparasite (Cachon, 1964) that uses oxygen throughout its life cycle (Farhat et al., 2018) and requires light in order to produce high numbers of infective dinospores (Figures 1A,B). Moreover, our results suggest that the parasite has access to oxygen, ATP, NADPH and other metabolites such as carbohydrates, from the host mitochondrion and chloroplast which remain functional during most of its intracellular stages of development. This suggest that the physiological stress of infection induced by *Amoebophrya* sp. A120 does not impact the functioning of the host organelles, a feature also reported in symbiotic dinoflagellates of collodarian where the chloroplast lasts under thermal stress until the very last stages of the cell lysis (Villar et al., 2018).

Frequently referred to as kleptochloroplastidy, plastid retention involves a modified grazing behavior where the predator sequestrates functional chloroplasts from its prey for a variable period of time (Johnson, 2011). Kleptochloroplastidy has been previously described in at least thirteen dinoflagellates genera (Stoecker et al., 2009). The stability of transient plastids may vary considerably: kleptochloroplastids of *Nusuttodinium* spp. and *Pfisteria piscicida* remain photosynthetically active for only a few days, whereas those of the genus *Dinophysis* are active over multiple cell divisions (Stoecker et al., 2009; Park et al., 2014). The organellar retention strategy observed in *Amoebophrya* lasts throughout the whole infection, a process similar to that used by the marine cyanophages infecting picocyanobacteria of the genera *Prochlorococcus* and *Synechococcus*. These viruses take control of the photosynthetic apparatus of their host during the infection (Dammeyer et al., 2008) by hijacking the transcriptional machinery in order to overexpress some photosynthetic proteins encoded in the viral genome, such as encoding the PSII core subunit PsbA (D1) (Mann et al., 2003; Lindell et al., 2005; Sharon et al., 2009). This strategy is so efficient that 60% of the *psbA* genes identifiable by barcoding at the ocean surfaces are thought to be of phage origin (Sharon et al., 2007). *Amoebophrya* strains lack the vestigial plastid-like structure found in other non-photosynthetic apicomplexans and the genomes of the sequenced strains are devoid of plastidial related genes (John et al., 2019). These suggest that *Amoebophrya* parasites are unable to take control of the host photosynthesis apparatus like cyanophages, but rather benefits passively from it. Given that organellar retention does not necessarily imply that plastids are actively maintained by the host, our results suggest that *Amoebophrya* sp. A120 performs a new form of “extra-cellular” organellar retention (klepto-organelly) involving both the chloroplast (kleptochloroplast) and potentially the mitochondrion (kleptomitochondrion) of its host. This is likely done by specifically avoiding the digestion of the host organelles during the parasite intracellular development, a process analogous to what happens during kleptoplastidy described in other protists/animals (Johnson, 2011). Comparison with other *Amoebophrya* strains digesting their host chloroplasts earlier during infection (Park et al., 2002b) may help understanding some of the mechanisms underlying organellar retention by the A120 strain.

## Data Availability Statement

The datasets generated for this study can be found in the online repositories. The names of the repository/repositories and accession number(s) can be found in the article/Supplementary Material.

## Author Contributions

LG, CS, and EK conceived this study. LV-S, JM, EB, DM, CS, and CA-S performed the physiological analyses. JS maintained cultures and performed reanalyses of the infection patterns. EK, BP, SF, BN, and EC performed the genetic analyses. CA-S performed statistical analyses. EK, LG, CS, and CA-S wrote the manuscript. All authors edited and approved the final version of this manuscript.

## Conflict of Interest

The authors declare that the research was conducted in the absence of any commercial or financial relationships that could be construed as a potential conflict of interest.

## Footnotes

1 http://roscoff-culture-collection.org

2 https://github.com/TransDecoder/TransDecoder/wiki

3 http://hmmer.org/

4 http://www.cbs.dtu.dk/services/TMHMM/

5 https://github.com/Trinotate/Trinotate.github.io/wiki

6 http://www.genome.jp/kegg/pathway.html

7 http://staden.sourceforge.net/

8 http://web.expasy.org/translate/

9 http://tree.bio.ed.ac.uk/software/figtree/

10 https://services.healthtech.dtu.dk/

11 http://application.sb-roscoff.fr/blast/hapar/

12 https://www.r-project.org/

## Supplementary Material

Supplementary Figure 1 Workflow of the RNA-seq analyses used in this study. The workflow was divided into three “steps” for clarity. See the “Materials and Methods” section for more details.

Supplementary Figure 2 Pigment variations in the phototrophic dinoflagellate *Scrippsiella acuminata* ST147 infected by the Syndiniales *Amoebophrya* sp. A120. **(A)** Variations of the relative natural chl *a* cell fluorescence measured by flow cytometry and **(B–F)** pigment content relative to chl *a* measured by HPLC in healthy (filled circles) and infected (empty circles) cultures under continuous light and expressed as a percentage of the values recorded at T0. Error bars represent standard deviation.

Supplementary Figure 3 Reconstruction of the mitochondrial electron transfer chain of the oxidative phosphorylation (OXPHOS) pathway in the photosynthetic dinoflagellate *Scrippsiella acuminata* ST147, highlighting the list of genes used in the differential expression analysis. Complexes II–V are identified following the nomenclature of the “Oxidative phosphorylation” (ko00190; OXPHOS) pathway defined by the KEGG Pathway database (http://www.genome.jp/kegg/pathway.html). All genes are encoded in the nucleus except for those in bold-italic (*cob*, *cox1*, and *cox3*) that are encoded in the mitochondrial DNA (mtDNA). Underlined genes harbor a transfer peptide at the N-terminus of the protein. Blue arrows represent the direction of electron transfer in the ETC; red arrows the flow of protons. Red dashed-delimited squares are enzymatic reactions belonging to other pathways that participate in mitochondrial electron transfer. Shaded gene illustrates a putative location. ICAP2 and ICAP18 genes are unconventional subunits identified in the mitochondrion of *Toxoplasma gondii*. Q represents the ubiquinone and its biosynthesis pathway was not included in this study. The full list of genes is found in Supplementary Table S1 and sequences can be downloaded from the *Amoebophrya* genome website (http://application.sb-roscoff.fr/project/amoebophrya).

Supplementary Figure 4 Reconstruction of the chloroplastidial electron transfer chain of the light phase of photosynthesis pathway in the photosynthetic dinoflagellate *Scrippsiella acuminata* ST147, highlighting the list of genes used in the differential expression analysis. Peridin-Chlorophyll binding Proteins (P), photosystem II and I antenna (LHC-like) proteins collect photons and transfer their energy to reaction centers to create the photosynthetic electron transport through a chain of several electron acceptors. Protein complexes are identified following the nomenclature of the “Photosynthesis” (ko00195 and ko00195) pathways defined by the KEGG Pathway database (http://www.genome.jp/kegg/pathway.html). In *Scrippsiella acuminata* ST147, the genes encoding most of these proteins are located in the nucleus except for those mentioned in italic (*psbA-E*, *petB*, *petD*, *psaA-B*, *atpF1A-B*), which are encoded in the chloroplast DNA (cpDNA). Underlined genes harbor a transfer and/or a signal peptide at the N-terminus of the protein. Blue arrows represent the direction of electron transfer in the ETC; red arrows the flow of protons. Shaded gene illustrates a putative location. The gene encoding the *psbI* subunit (in yellow) of PSII normally encoded by the cpDNA was not recovered. PQ represents the plastoquinone whose biosynthesis pathway was not included in this study. The full list of genes is found in see Supplementary Table S2 and sequences can be downloaded from the *Amoebophrya* genome website (http://application.sb-roscoff.fr/project/amoebophrya).

Supplementary Figure 5 Expression patterns of genes whose products are involved in the electron transfer chain of the mitochondrial OXPHOS pathway in the phototrophic dinoflagellate host *Scrippsiella acuminata* ST147 infected by the Syndiniales *Amoebophrya* sp. A120 over a full 36 h infection cycle. Genes have been grouped depending on their genome location (mitochondrion or nucleus) and membrane complex (see Supplementary Figure S3 for more details). Horizontal lines and error bars represent averages and standard deviation, respectively.

Supplementary Figure 6 Expression patterns of genes whose products are involved in the electron transfer chain of the chloroplast photosynthesis pathway in the phototrophic dinoflagellate host *Scrippsiella acuminata* infected by the Syndiniales *Amoebophrya* sp. strain A120 over a full 36 h infection cycle. Genes have been grouped depending on their genome location (chloroplast or nucleus) and membrane complex (see Supplementary Figure S4 for more details). Horizontal lines and error bars represent averages and standard deviation, respectively.

Supplementary Table 1 List of genes involved in the electron transfer chain of the mitochondrial OXPHOS pathway in the phototrophic dinoflagellate host *Scrippsiella acuminata* ST147 used in this study.

Supplementary Table 2 List of genes involved in the electron transfer chain of the chloroplast photosynthesis pathway in the phototrophic dinoflagellate host *Scrippsiella acuminata* ST147 used in this study.

Supplementary Video 1 Culture of *Scrippsiella acuminata* ST147 infected by *Amoebophrya* sp. A120 where both healthy (predominantly red due to natural chlorophyll *a* fluorescence) and infected cells (predominantly green due to *Amoebophrya* natural green fluorescence) can be seen swimming actively.
