# Supplementary figures and images for: Dinoflagellate Host Chloroplasts and Mitochondria Remain Functional During Amoebophrya Infection
Source: Front Microbiol. 2020 Dec 18;11:600823. doi: 10.3389/fmicb.2020.600823 (PMC7793755; doi:10.3389/fmicb.2020.600823)

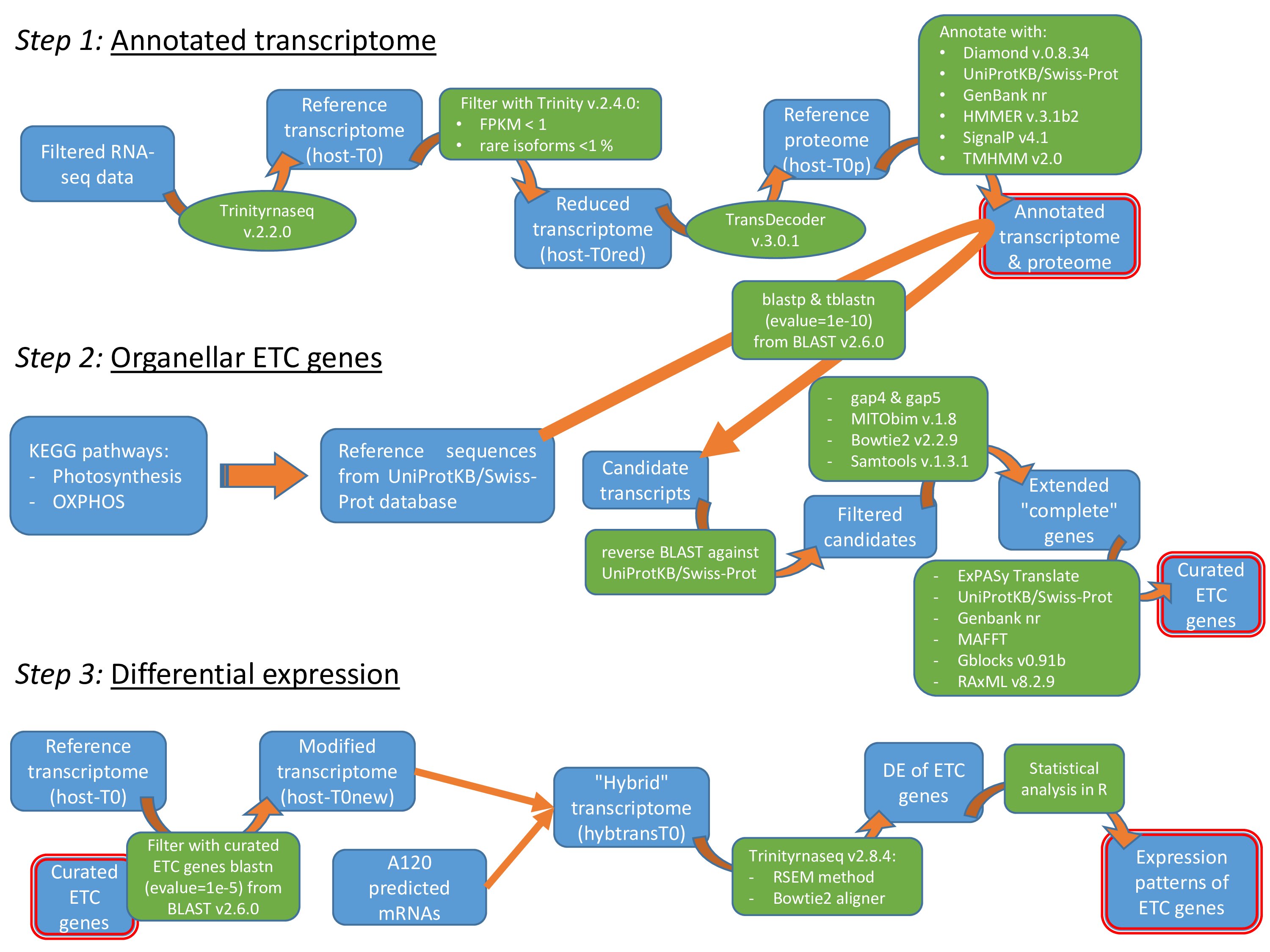

Supplement: Supplementary Figure 1 — Workflow of the RNA-seq analyses used in this study. The workflow was divided into three “steps” for clarity. See the “Materials and Methods” section for more details. [file Image_1.JPEG]

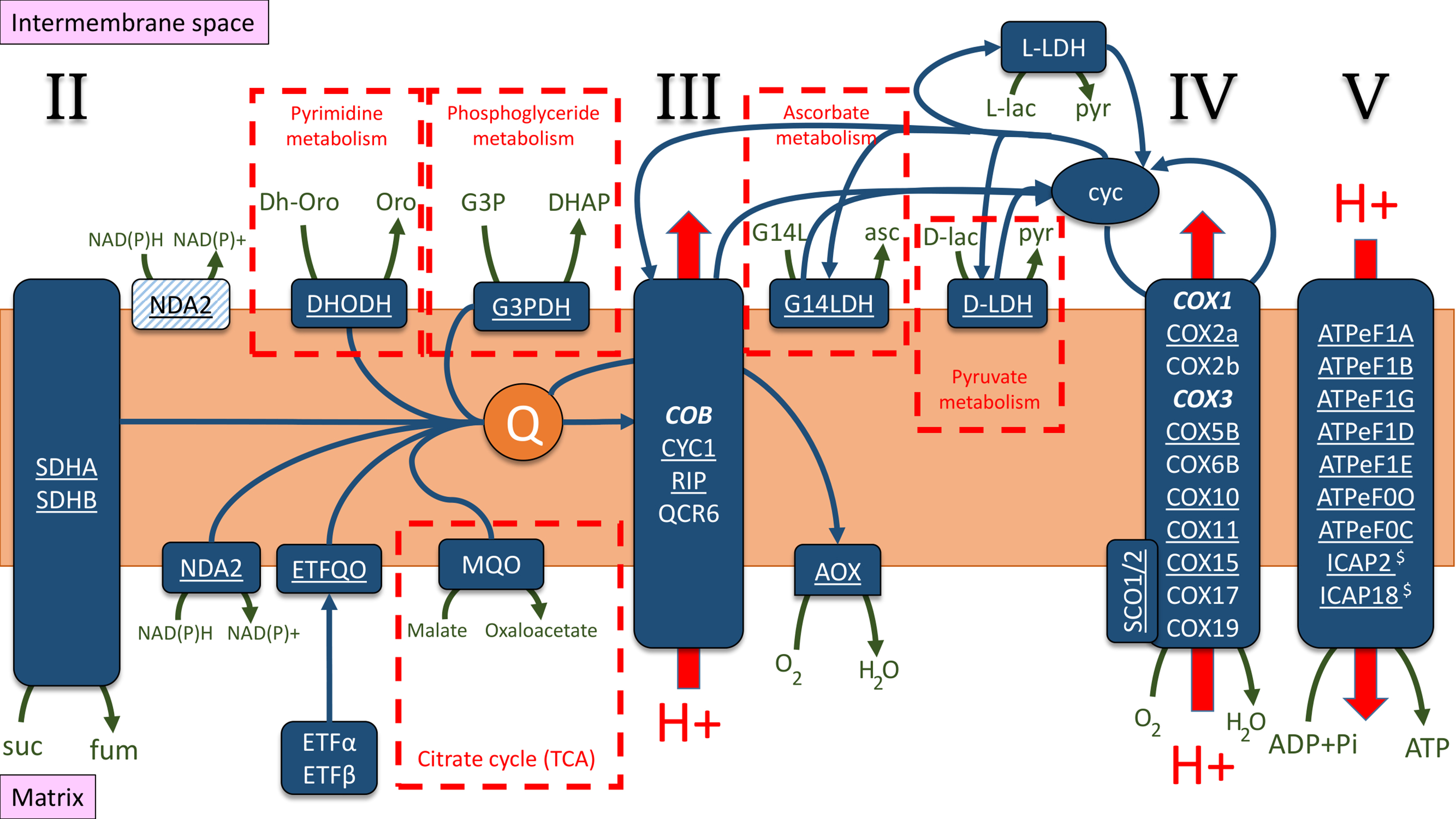

Supplement: Supplementary Figure 2 — Pigment variations in the phototrophic dinoflagellate Scrippsiella acuminata ST147 infected by the Syndiniales Amoebophrya sp. A120. (A) Variations of the relative natural chl a cell fluorescence measured by flow cytometry and (B–F) pigment content relative to chl a measured by HPLC in healthy (filled circles) and infected (empty circles) cultures under continuous light and expressed as a percentage of the values recorded at T0. Error bars represent standard deviation. [file Image_2.jpg]

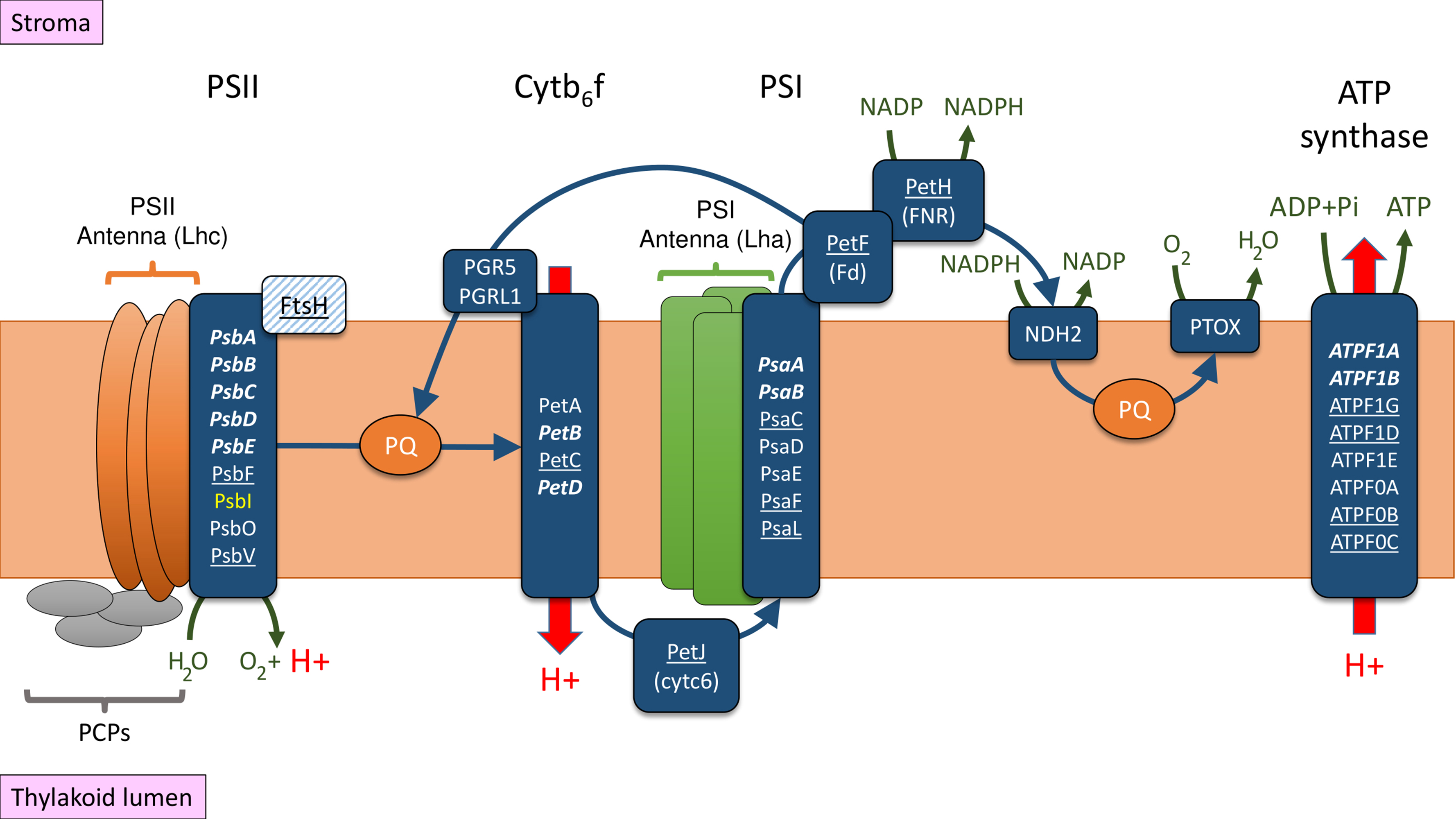

Supplement: Supplementary Figure 3 — Reconstruction of the mitochondrial electron transfer chain of the oxidative phosphorylation (OXPHOS) pathway in the photosynthetic dinoflagellate Scrippsiella acuminata ST147, highlighting the list of genes used in the differential expression analysis. Complexes II–V are identified following the nomenclature of the “Oxidative phosphorylation” (ko00190; OXPHOS) pathway defined by the KEGG Pathway database (http://www.genome.jp/kegg/pathway.html). All genes are encoded in the nucleus except for those in bold-italic (cob, cox1, and cox3) that are encoded in the mitochondrial DNA (mtDNA). Underlined genes harbor a transfer peptide at the N-terminus of the protein. Blue arrows represent the direction of electron transfer in the ETC; red arrows the flow of protons. Red dashed-delimited squares are enzymatic reactions belonging to other pathways that participate in mitochondrial electron transfer. Shaded gene illustrates a putative location. ICAP2 and ICAP18 genes are unconventional subunits identified in the mitochondrion of Toxoplasma gondii. Q represents the ubiquinone and its biosynthesis pathway was not included in this study. The full list of genes is found in Supplementary Table S1 and sequences can be downloaded from the Amoebophrya genome website (http://application.sb-roscoff.fr/project/amoebophrya). [file Image_3.jpg]

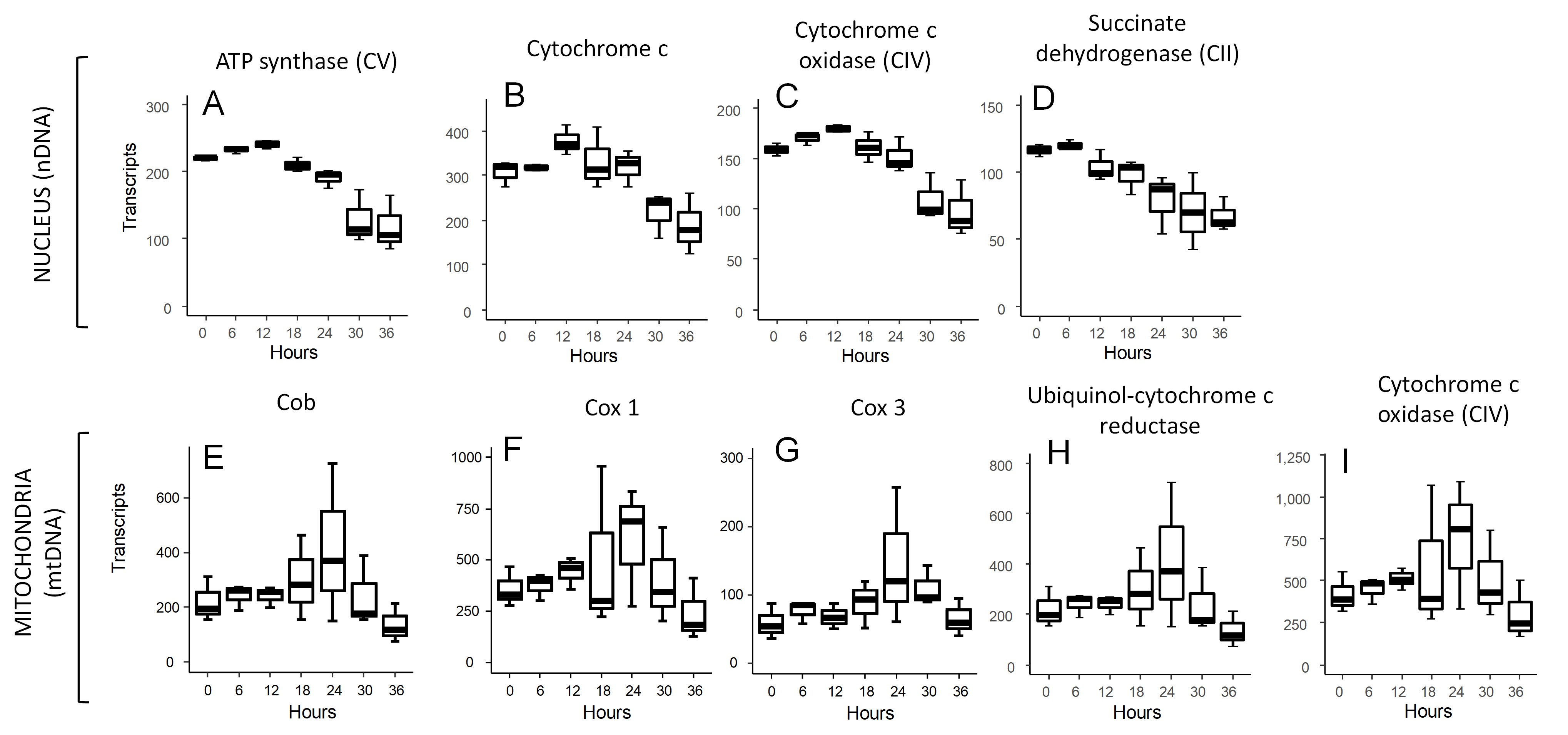

Supplement: Supplementary Figure 4 — Reconstruction of the chloroplastidial electron transfer chain of the light phase of photosynthesis pathway in the photosynthetic dinoflagellate Scrippsiella acuminata ST147, highlighting the list of genes used in the differential expression analysis. Peridin-Chlorophyll binding Proteins (P), photosystem II and I antenna (LHC-like) proteins collect photons and transfer their energy to reaction centers to create the photosynthetic electron transport through a chain of several electron acceptors. Protein complexes are identified following the nomenclature of the “Photosynthesis” (ko00195 and ko00195) pathways defined by the KEGG Pathway database (http://www.genome.jp/kegg/pathway.html). In Scrippsiella acuminata ST147, the genes encoding most of these proteins are located in the nucleus except for those mentioned in italic (psbA-E, petB, petD, psaA-B, atpF1A-B), which are encoded in the chloroplast DNA (cpDNA). Underlined genes harbor a transfer and/or a signal peptide at the N-terminus of the protein. Blue arrows represent the direction of electron transfer in the ETC; red arrows the flow of protons. Shaded gene illustrates a putative location. The gene encoding the psbI subunit (in yellow) of PSII normally encoded by the cpDNA was not recovered. PQ represents the plastoquinone whose biosynthesis pathway was not included in this study. The full list of genes is found in see Supplementary Table S2 and sequences can be downloaded from the Amoebophrya genome website (http://application.sb-roscoff.fr/project/amoebophrya). [file Image_4.jpg]

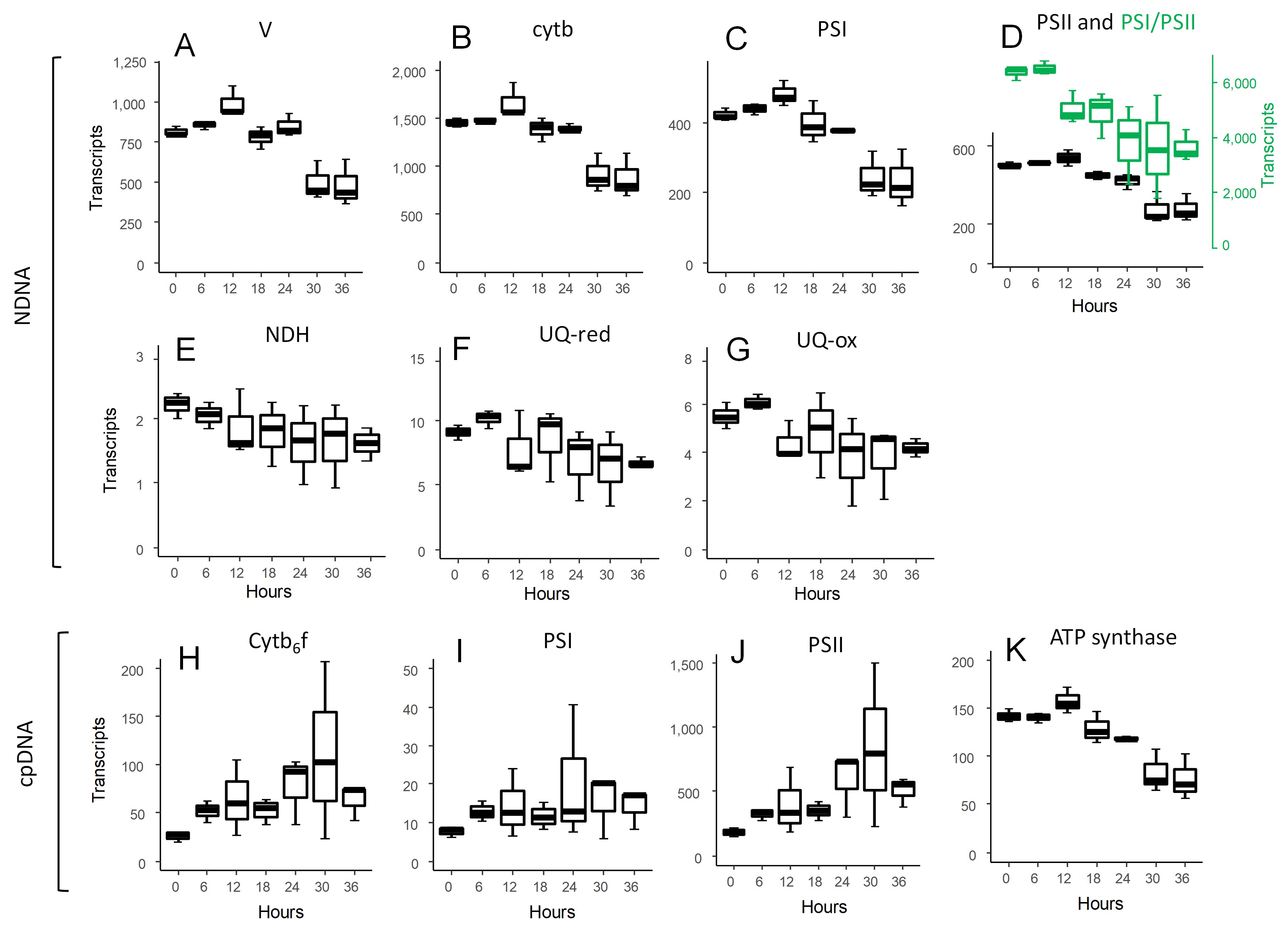

Supplement: Supplementary Figure 5 — Expression patterns of genes whose products are involved in the electron transfer chain of the mitochondrial OXPHOS pathway in the phototrophic dinoflagellate host Scrippsiella acuminata ST147 infected by the Syndiniales Amoebophrya sp. A120 over a full 36 h infection cycle. Genes have been grouped depending on their genome location (mitochondrion or nucleus) and membrane complex (see Supplementary Figure S3 for more details). Horizontal lines and error bars represent averages and standard deviation, respectively. [file Image_5.JPEG]

**A**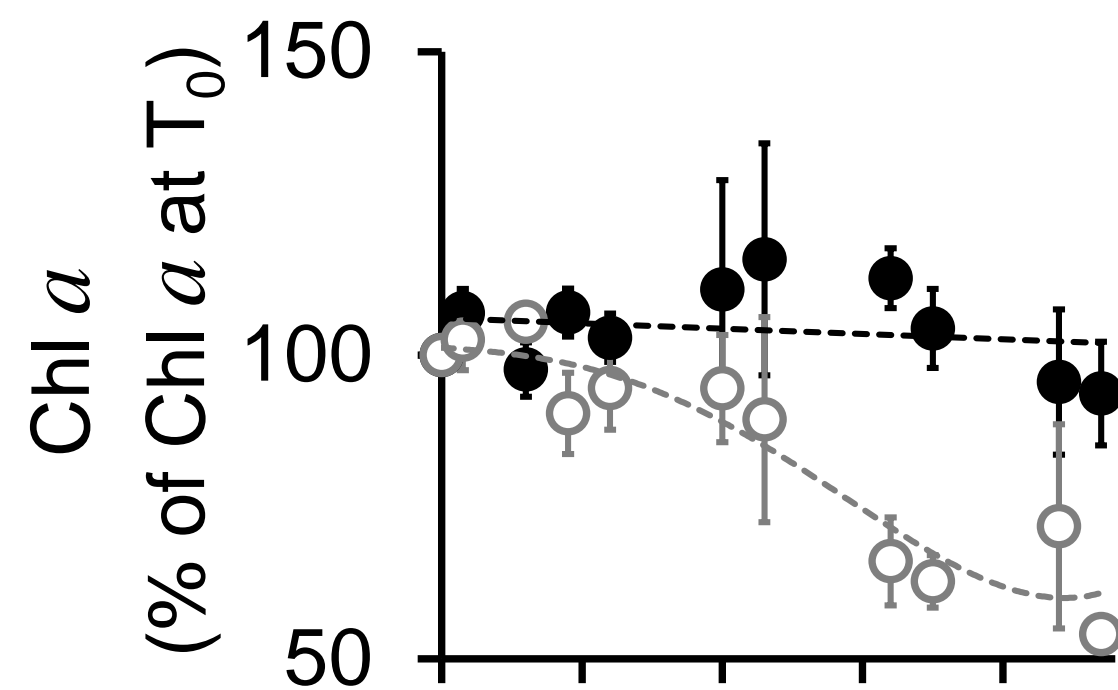**B**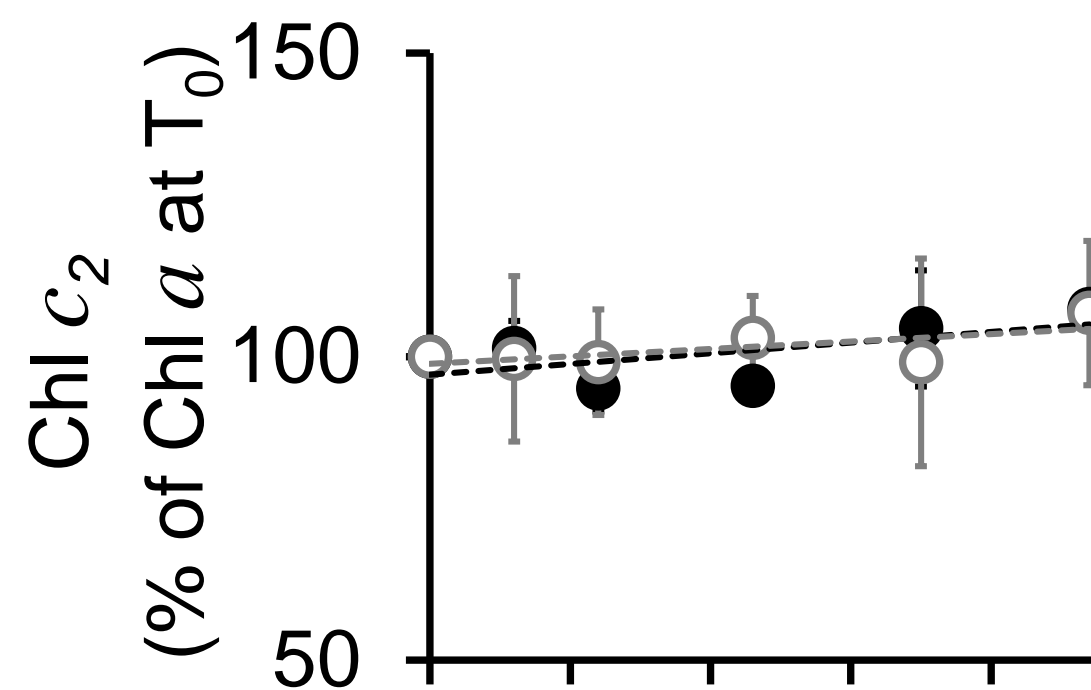**C**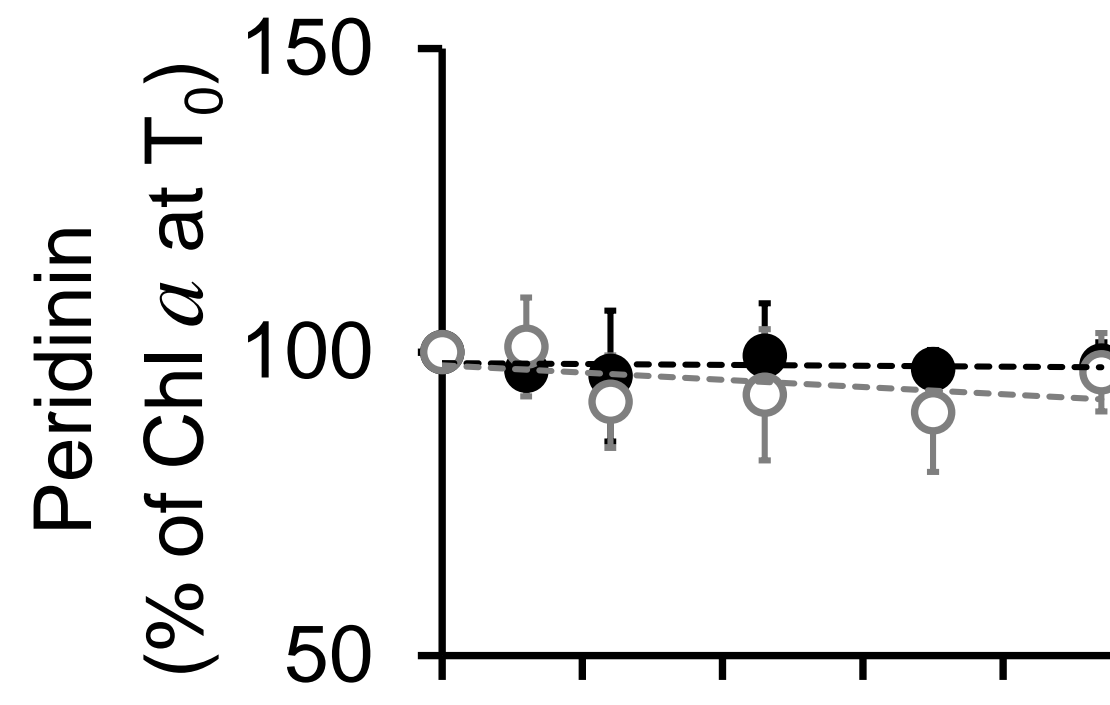**D**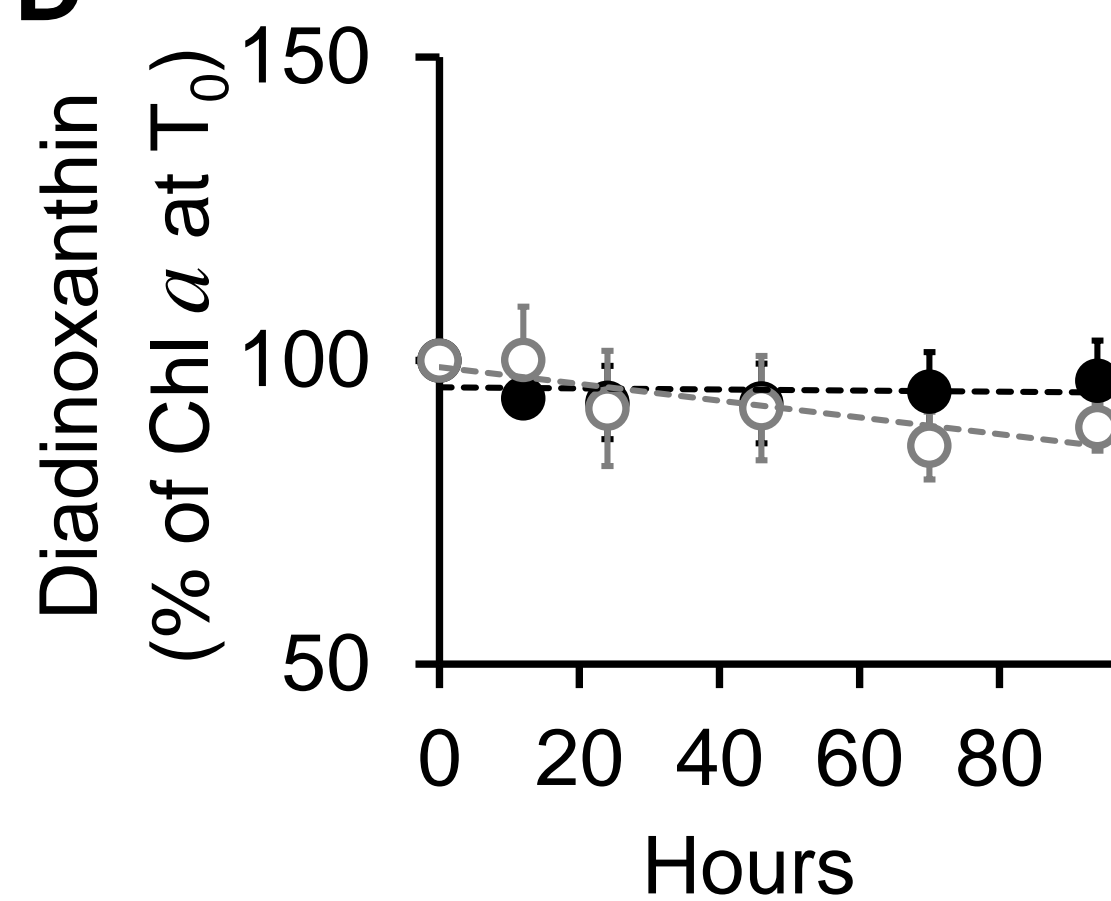**E**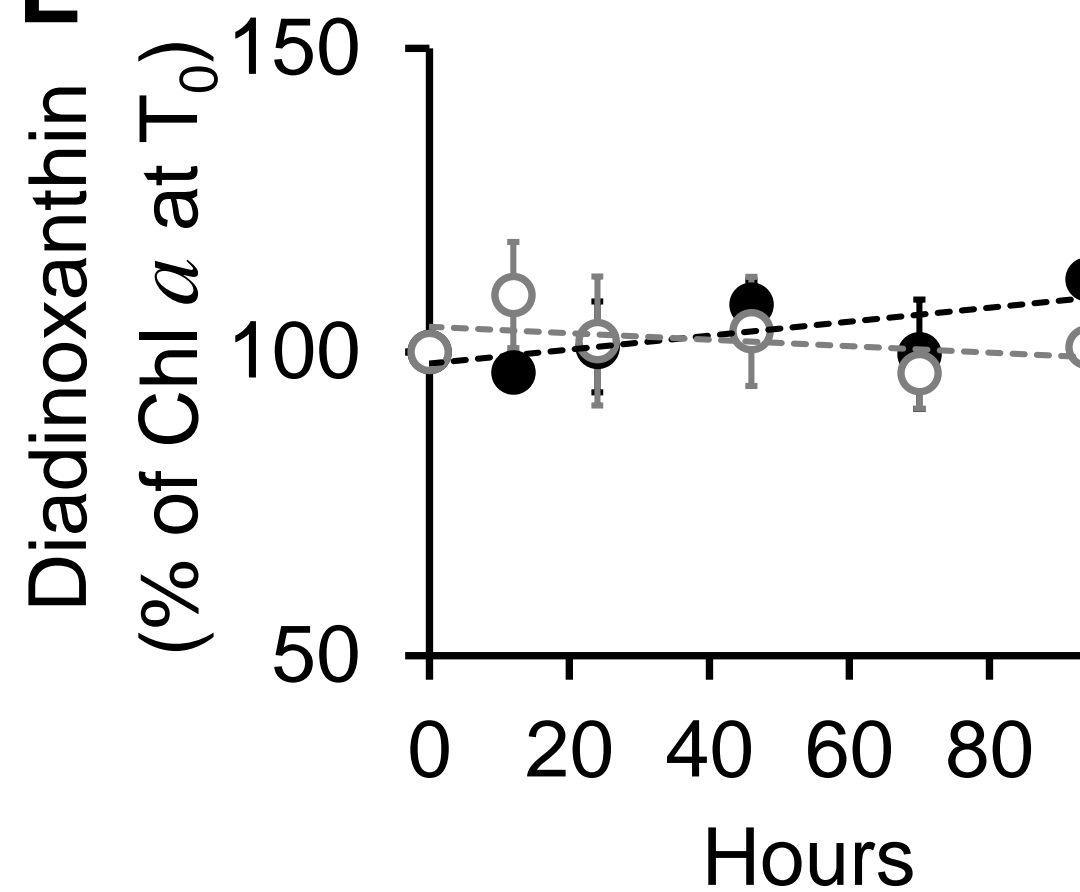**F**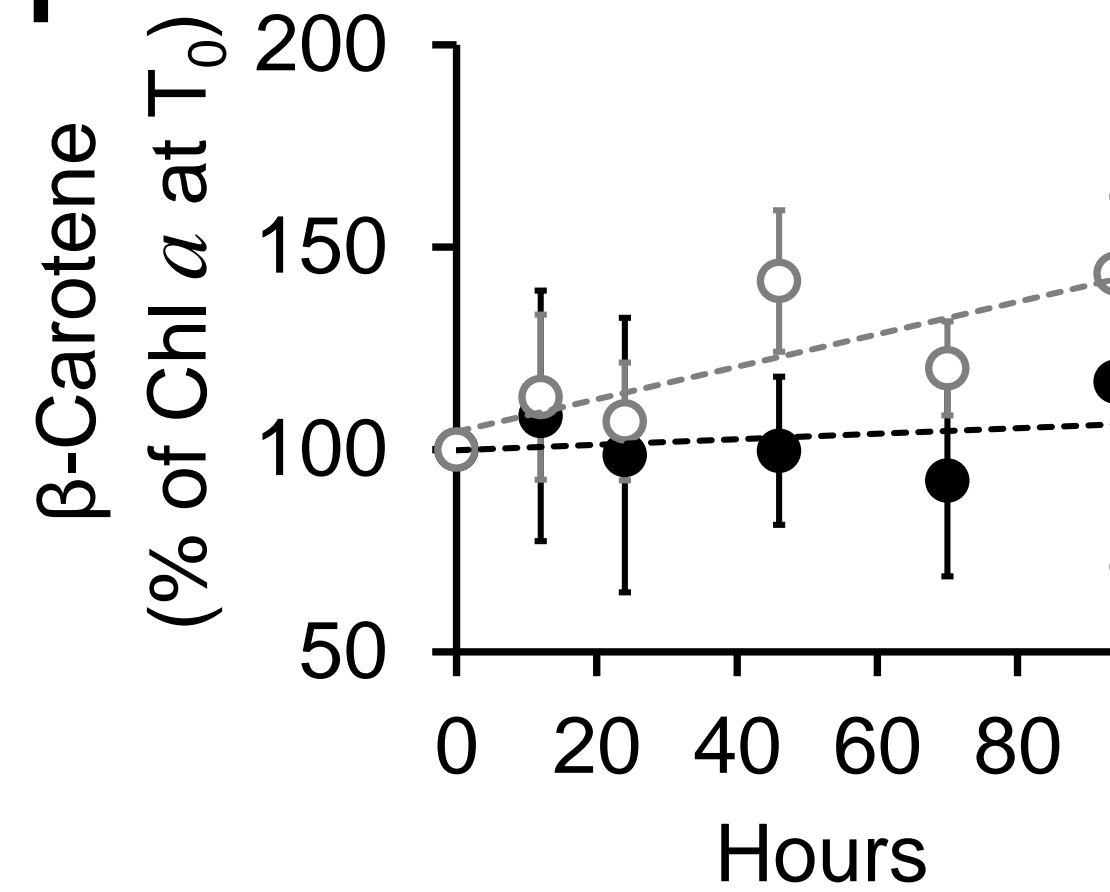

Supplement: Supplementary Figure 6 — Expression patterns of genes whose products are involved in the electron transfer chain of the chloroplast photosynthesis pathway in the phototrophic dinoflagellate host Scrippsiella acuminata infected by the Syndiniales Amoebophrya sp. strain A120 over a full 36 h infection cycle. Genes have been grouped depending on their genome location (chloroplast or nucleus) and membrane complex (see Supplementary Figure S4 for more details). Horizontal lines and error bars represent averages and standard deviation, respectively. [file Data_Sheet_1.PDF]
